# Collagen fibrillogenesis: fibronectin, integrins, and minor collagens as organizers and nucleators

**DOI:** 10.1016/j.ceb.2008.06.008

**Published:** 2008-10

**Authors:** Karl E Kadler, Adele Hill, Elizabeth G Canty-Laird

**Affiliations:** Wellcome Trust Centre for Cell-Matrix Research, University of Manchester, Faculty of Life Sciences, Michael Smith Building, Oxford Road, Manchester M13 9PT, United Kingdom

## Abstract

Collagens are triple helical proteins that occur in the extracellular matrix (ECM) and at the cell–ECM interface. There are more than 30 collagens and collagen-related proteins but the most abundant are collagens I and II that exist as *D*-periodic (where *D* = 67 nm) fibrils. The fibrils are of broad biomedical importance and have central roles in embryogenesis, arthritis, tissue repair, fibrosis, tumor invasion, and cardiovascular disease. Collagens I and II spontaneously form fibrils *in vitro*, which shows that collagen fibrillogenesis is a selfassembly process. However, the situation *in vivo* is not that simple; collagen I-containing fibrils do not form in the absence of fibronectin, fibronectin-binding and collagen-binding integrins, and collagen V. Likewise, the thin collagen II-containing fibrils in cartilage do not form in the absence of collagen XI. Thus, *in vivo*, cellular mechanisms are in place to control what is otherwise a protein self-assembly process. This review puts forward a working hypothesis for how fibronectin and integrins (the organizers) determine the site of fibril assembly, and collagens V and XI (the nucleators) initiate collagen fibrillogenesis.

## Introduction

A crucial event in limb development occurs soon after mesenchymal condensation when collagen fibrils begin to appear in the extracellular matrix (ECM) of rudimentary tendons, ligaments, bones, and joints. The fibrils increase in size and abundance during embryonic and postnatal development until they become the most abundant structural element in adult tissues. Our understanding of how collagen fibrillogenesis is initiated and regulated is limited, and this represents a major setback to attempts in regenerative medicine to replace ECM-rich organs and tissues lost to disease or trauma.

## The collagen fibril enigma

Collagen I fibril formation *in vitro* is a straightforward example of entropy-driven protein self-assembly/polymerization and has a critical concentration of ∼1 nM at 37 °C (see [1] and references therein). Early experiments showed that collagen I could be extracted from tissues in dilute acidic solutions or high-salt buffers and reconstituted into fibrils by neutralizing and/or warming the solutions [2]. Subsequent studies identified binding sites on the collagen monomers for fibril formation [3]. The case for support for collagen self-polymerization is strengthened by the fact that collagen is synthesized as a soluble precursor, procollagen, containing N-terminal and C-terminal propeptides. The propeptides are cleavable by procollagen N-proteinases and C-proteinases (for review see [4] and references therein). Moreover, cleavage of the C-propeptides by the BMP-1/tolloid family of metalloproteinases is sufficient to initiate collagen fibrillogenesis *in vitro* [5]. These studies provide unequivocal proof that collagen fibril formation can occur readily in the absence of cells. Consequently, the absolute requirement for noncollagenous molecules to initiate collagen fibrillogenesis *in vivo* is mystifying.

The answer to this riddle most probably lies in the fact that fibrillar collagens have ∼50 known binding partners *in vivo* [6]. This number of binding partners is presumably required to generate the diversity of fibril patterns, which range from parallel bundles in tendon and ligament, to orthogonal lattices in cornea, and interlocking weaves in blood vessels, skin, and bone. On the contrary, having a large number of binding partners can spell disaster for a protein self-assembly/polymerization process. Purified collagen spontaneously assembles into fibrils *in vitro* because collagen molecules are free to bind to other collagen molecules, and only collagen molecules. *In vivo*, however, the situation is very different. Faced with so many potential-binding partners, collagen molecules might easily be sequestered into dead-end molecular interactions, which would lower the effective concentration of collagen monomers available to form fibrils. In the remainder of the review, we explore the possibility that cells use collagen V and XI to nucleate collagen fibrils, and fibronectin (FN) and integrins to specify their site of assembly. By localizing fibril formation to the plasma membrane the cell maintains tight regulatory control of collagen fibrillogenesis, which is clearly essential for the formation of long-range packing assemblies of collagen fibrils in different tissues.

## The organizers — fibronectin and integrins

Several molecules are involved in the biosynthesis of collagen. These include ER-resident glycosyltransferases and isomerases as well as the collagen-specific molecular chaperone Hsp47. Furthermore, the ADAMTS procollagen N-proteinases and BMP-1/tolloid C-proteinases are essential for the conversion of procollagen to a fibrillogenesis-competent molecule. However, leaving aside these important biosynthetic proteins, FN and integrins appear to be essential for the formation of collagen fibrils by cultured cells (see below) and mesenchymal embryonic cells *in vivo* (Hill and Kadler, in preparation). The detailed mechanism of FN-mediated and integrin-mediated collagen fibrillogenesis has not been elucidated.

FN is secreted as a disulfide-bonded dimer having three types of repeating modules (i.e. type I, II, and III repeats) that mediate interactions with cells, ECM components (including collagen [7]) and other FN molecules (for review see [8]). The cognate FN–collagen-binding sites are located at the 3/4–1/4 mammalian collagenase cleavage site on collagen [9,10] and within a region near the N-terminus of FN that contains type I and II module repeats [11–15].

FN polymerization is a cell-dependent process that requires direct interactions with integrin receptors [16–19]. Once engaged with integrins, FN undergoes a conformation change that exposes a cryptic site for FN–FN polymerization [20,21]. Seminal studies showed antibody binding to the collagen-binding site on FN-inhibited collagen fibrillogenesis [22]. Interestingly, there was a reciprocal dependence of FN fibril assembly and collagen fibril assembly; fibroblasts from the Mov13 mouse (in which the COL1A1 gene is inactivated by retroviral insertion in an intron) establish a sparse FN network, which can be restored by transfection of the COL1A1 gene [23].

The requirement of FN for collagen fibril assembly is not restricted to fibroblasts. Collagen fibril assembly by vascular smooth muscle cells was inhibited by an anti-α2β1 integrin antibody and accelerated by an α2β1 integrin antibody that stimulates a high-affinity binding state of the integrin [24]. In the same study, newly assembled collagen fibrils were found to colocalize with newly assembled FN fibrils. Also, the inhibition of FN assembly with an anti-α5β1 integrin antibody completely inhibited collagen assembly. Of further interest, disruption of smooth muscle cell actin microfilaments using cytochalasin resulted in almost no collagen fibril assembly on the cells [24].

It seems probable, therefore, that FN fibril assembly and collagen fibril assembly have mechanistic elements in common, involving functional integration of the cytoskeleton with plasma membrane-located integrins. In the case of FN, an α5β1 integrin-induced conformational change is necessary to promote fibrillogenesis. It is less clear how integrins and FN catalyze collagen fibrillogenesis. A tantalizing possibility is that FN and/or integrins induce a conformational change in collagen to accelerate fibrillogenesis. Alternatively, collagen molecules might be brought into close proximity on the surface of FN fibrils or by the engagement with integrins (e.g. α2β1).

The site of collagen–FN–integrin interactions during collagen fibrillogenesis is unknown. Procollagen and FN have been colocalized in the secretory pathway of cultured fibroblasts [25], therefore it is possible that FN–procollagen interactions are established before the molecules are secreted. Furthermore, procollagen can be cleaved to collagen within the cell [26–28], which might also mean that FN(*monomer*)–collagen(*monomer*) interactions occur before secretion. Additional research is needed to determine if FN–procollagen–collagen complexes are copresented to integrins at the plasma membrane or within the secretory pathway. Collagen fibril assembly in embryonic development occurs in recesses of the plasma membrane [29,30^•^], and newly formed collagen fibrils occur in plasma membrane protrusions called fibripositors [26]. Further studies are needed to determine if plasma membrane recesses and fibripositors are special sites of FN and collagen fibril assembly.

## The nucleators — collagens V and XI

The Ehlers-Danlos syndrome (EDS) is characterized by joint hypermobility and skin laxity and can be caused by mutations in a variety of ECM genes including collagen V and tenascin-X. In elegant studies of EDS, Wenstrup *et al.* showed that collagen V is essential for the assembly of collagen I-containing fibrils *in vivo* [31^••^,32]. Collagen V codistributes with collagen I [33] and mice lacking collagen V die at embryonic day 10 because of cardiovascular failure associated with a lack of collagen fibrils in the mesenchyme. Heterozygous mice are viable and have a 50% reduction in fibril number and dermal collagen content, caused either by a direct or indirect consequence of half normal levels of collagen V protein. This result is entirely consistent with a study of fibroblasts from an EDS-affected individual with COL5A1 haploinsufficiency in which the total incorporation of collagen into collagen fibrils was reduced by half and was associated with a proportionate decrease in fibril number [32]. Collagen V contains B clade polypeptide chains having extended thrombospondin-like and variable domains at the N-termini [34]. Electron microscope studies have shown that the triple helical domain of collagen V is buried in the fibril with the N-terminal domains at the fibril surface. The prime location of collagen V at the fibril core and its persistence in the final fibril indicates that this collagen is important in nucleating collagen I-containing fibrils *in vivo* [35^•^,36,37].

**Figure 1:**
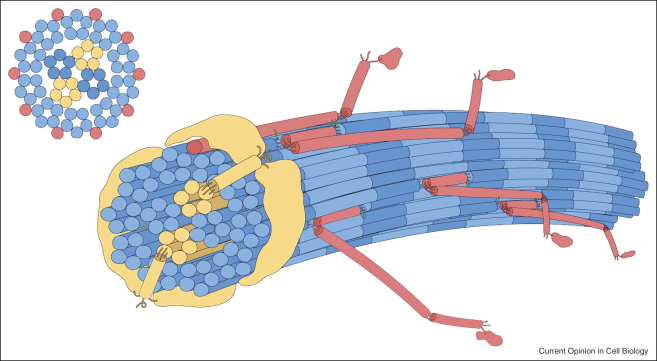
Schematic of the 10 + 4 microfibril structure of a thin cartilage collagen fibril. A pair of collagen XI microfibrils comprise half of a 4 microfibril core surrounded by 10 microfibrils at the surface. The collagen XI/IX/II assembly is a crosslinked heteropolymer, as is V/I, and is an important component of the fibril assembly mechanism. Blue: collagen II molecules; yellow: collagen XI molecules; red: collagen IX molecules. The N-terminal thrombospondin-like domains of collagen XI (yellow) are shown extending from the core microfibrils onto the fibril surface (model kindly provided by Dr David Eyre, University of Washington, Seattle).

Collagen XI shares structural homology with collagen V and appears to have a similar nucleating function. Together with collagens II and IX, collagen XI forms *D*-periodic heterotypic fibrils in cartilage [38–43]. Cartilage fibrils exist in distinct populations of ‘thin’ (16-nm diameter) and ‘thick’ (∼40-nm diameter) fibrils and collagen XI is found exclusively in the thin fibrils [39]. Electron microscopy studies show that thin cartilage fibrils are constructed from 14 tilted 4-nm-diameter microfibrils, in a 10 + 4 arrangement, with collagen XI located in microfibrils at the core of the fibril [35^•^] (Figure 1).

These observations are relevant to the *cho*/*cho* mouse and human osteochondrodysplasias caused by mutations in the genes for collagen XI. Autosomal recessive chondrodysplasia (*cho*) affects the cartilage in limbs, ribs, mandibles, and trachea and is accompanied by the absence of thin fibrils [44]. The causative mutation in the *cho*/*cho* mouse is localized in the gene encoding the α1(XI) chain of collagen XI [45], resulting in alternative assemblies of collagen XI molecules in the ECM [46^••^]. The absence of thin cartilage fibrils in the *cho*/*cho* mouse suggests that collagen XI is required to nucleate the assembly of the thin fibrils. Like the thin fibrils in cartilage, the thin heterotypic collagen I/V fibrils in the corneal stroma also have a microfibrillar substructure [47].

## Regulators

Numerous proteins, glycoconjugates, and small molecules have been shown to influence the rate of assembly, size, and structure of collagen fibrils formed *in vitro*. In some instances mouse models have been generated for *in vivo* analysis. The list is extensive and a comprehensive review of these proteins is outside the scope of this article. However, the molecules that have attracted most attention are the N-propeptides of collagen I (see [48] and references therein), crosslinking enzymes such as lysyl oxidase (see [49] and references therein), tenascin-X, thrombospondin 2 [50,51], cartilage oligomeric matrix protein (COMP) [52], matrilins [53], perlecan [54^•^], and the small leucine-rich proteoglycans decorin [55], biglycan [56], fibromodulin [57,58], and lumican [57,59,60].

Tenascin-X deserves a special mention because its level of expression is associated with the number of collagen fibrils, and its deficiency in humans is associated with EDS. The gene for tenascin-X was the first EDS gene not to encode a fibrillar collagen or procollagen processing enzyme [61]. Subsequent analysis of a tenascin-X murine model of EDS led to further surprising observations [62^••^]; mice lacking tenascin-X had collagen fibrils of normal size and shape but the packing density of the fibrils in the dermis was much reduced, leading to a 30% reduction in collagen content in skin. Interestingly, skin fibroblasts from the null animals had near normal collagen synthesis but a significant deficit in the amount of collagen deposited into insoluble matrix. This led to the conclusion that tenascin-X deficiency does not interfere with collagen synthesis or processing, but, rather, acts by regulating fibril assembly.

The current evidence suggests that tenascin-X regulates the spacing between fibrils by binding directly via its C-terminal fibrinogen-like domain and via its 10th and 11th FN type III repeats to decorin located on the surface of collagen fibrils ([63–65] and reviewed by [66]). Tenascin-X also accelerates collagen fibril formation *in vitro* [67] and has an additive effect on the rate of collagen fibril formation in the presence of collagen VI [68].

## Perspectives

**Figure 2:**
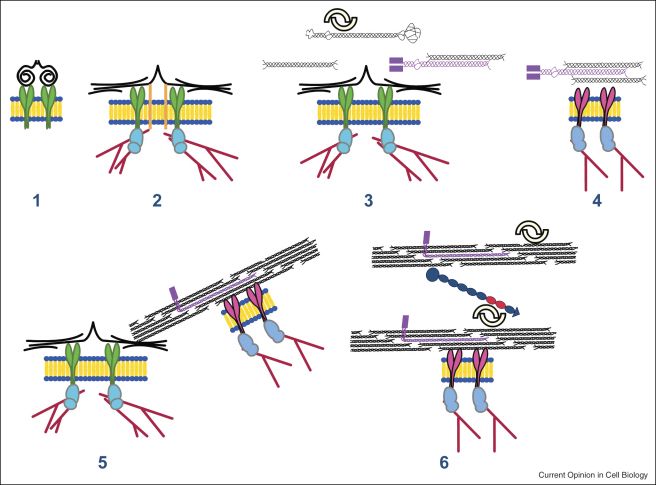
Hypothetical model of collagen fibril nucleation at the plasma membrane. (1) Dimeric FN (black) binds to α5β1 integrins (green). (2) Engagement of the integrin with the cytoskeleton (red lines) causes a conformational change in FN with subsequent fibril formation. Additional receptors (orange bars) bind FN. (3) Collagen I, procollagen I (black), and collagen V (purple) engage with FN at the fibril surface to facilitate collagen fibril formation. Decorin (interlocking dimers) shown bound to procollagen. (4) Activated collagen integrins (e.g. α2β1) bind collagen and induce a conformation change that facilitates fibril formation. (5) Collagen fibril formation at the cell surface. (6) Interactions between collagen fibrils (e.g. including tenascin-X and decorin) determine fibril diameter, organization, and spacing. Parts of the schematic are adapted from Mao and Schwarzbauer [8] and Bristow *et al.* [66].

The importance of collagen V, FN, α5β1, and α2β1 integrins in collagen fibrillogenesis implicates that these molecules are at a common membrane-located site for collagen fibril assembly. Figure 2 shows a theoretical scheme for collagen fibrillogenesis that incorporates what is currently known about the interaction of these molecules. It is presented here purely to stimulate discussion and to encourage new research directions. Integrin-mediated FN assembly is shown preceding collagen fibril assembly. However, copolymerization might occur, the order of assembly might be cell specific, and other (as yet unidentified) proteins are likely to be involved. Dimeric decorin is shown attached to the surfaces of collagen fibrils but this is not intended to exclude other small leucine-rich proteoglycans or to exclude a role for monomeric proteoglycans in collagen fibril assembly.

Further studies are needed to determine the high-resolution structure of collagen fibrils *in vivo* particularly in the context of understanding the molecular basis of tissue organization. Such structural studies should preferably be performed on hydrated fibrils (ideally *in situ*) to ensure that native structures and molecular interactions are maintained. Building on new knowledge of fibril structure, major advances should follow in understanding how fibrils interact with cells.

The crucial importance of the cytoskeleton and secretory pathway in collagen fibrillogenesis is exemplified in developing tendon in which newly formed fibrils occur in plasma membrane channels and fibripositors. However, progress in studying plasma membrane channels and fibripositors has been hampered by the lack of suitable cell culture systems; cells in monolayer do not have channels nor do they assemble an ECM of organized collagen fibrils. Recent studies show that three-dimensional tensioned fibrin gels are a suitable cell culture system for studying fibripositors and cell-mediated collagen fibrillogenesis [69^•^]. The use of three-dimensional cell culture systems in combination with molecular perturbation methods and correlative light-and-electron microscopy should lead to discoveries about the molecular mechanisms of FN–collagen–integrin-mediated ECM assembly.

## References and recommended reading

Papers of particular interest, published within the period of review, have been highlighted as:

• of special interest
•• of outstanding interest
